# The Association between the Risk of Premenstrual Syndrome and Vitamin D, Calcium, and Magnesium Status among University Students: A Case Control Study

**DOI:** 10.15171/hpp.2015.027

**Published:** 2015-10-25

**Authors:** Afsaneh Saeedian Kia, Reza Amani, Bahman Cheraghian

**Affiliations:** ^1^International Arvand Medical Sciences University, Abadan, Iran; ^2^Department of Nutrition, Diabetes Research Center, Jundishapur University of Medical Sciences, Ahvaz, Iran; ^3^Department of Epidemiology, Jundishapur University of Medical Sciences, Ahvaz, Iran

**Keywords:** Premenstrual Syndrome, Calcium, Magnesium, Vitamin D

## Abstract

**Background:** Premenstrual syndrome (PMS) is one of major health problems in childbearing age women. Herein, we compared the nutritional status of vitamin D, calcium (Ca) and magnesium (Mg) in young students affected by PMS with those of normal participants.

**Methods:** This study was conducted on 62 students aged 20‒25 yr in the city of Abadan (31 PMS cases and 31 controls). All participants completed four or more criteria according to the Utah PMS Calendar 3. Age, height, body mass index (BMI), serum Ca, Mg and vitamin D levels and a 24-hour food recall questionnaire were recorded.

**Results:** Vitamin D serum levels were lower than the normal range in the two groups. The odds ratios (CI 95%) of having PMS based on serum Ca and Mg concentrations were 0.81(0.67 – 0.89) and 0.86 (0.72 – 0.93), respectively. Based on serum levels, 855 of all participants showed vitamin D deficiency and more than one-third of the PMS cases were Mg deficient (P<0.05). In addition, there were signifi­cant differences in dietary intake of Ca and Mg, and potassium but not vitamin D in the two groups. Dietary intakes of Ca and Mg were quite below the recommendation in all participants.

**Conclusion:** Vitamin D, Ca and Mg nutritional status are compromised in PMS subjects. Because PMS is a prevalent health problem among young women, it merits more attention regarding improvement of their health and nutritional status.

## Introduction

 Conflicting advances to premenstrual health management have been related with a wide range of hygiene and psychosocial outcomes.^1^ Premenstrual syndrome (PMS) is a condition with a high prevalence in our modern society. It beings with reverent episodes of physical and psychological signs and symptoms that occur in luteal phase and over the onset of periodic hemorrhage it disappears.^2^ PMS syndrome has signs related to menstrual period of women. These signs included behavioral changes, increased appetite, depression and feeling tired seen in 3 out of 4 in women’s periodic cycles. Some women experience this syndrome in their 20s; however, others are involved in the late 30s and 40s.^3^ Both physical and psychological symptoms of PMS affect the health and quality of life in women.^4^

 Epidemiological studies have revealed that 75% of fertile women have mild to moderate symptoms. This condition has a predictable rate of involvement.^5^ Amongst them, 3-8% may present severe symptoms, which may hinder their daily activities.^6,7^ Although medicine has witnessed many achievements in this field in recent years, the etiology of PMS still remains uncertain.^8^

 Although there are many treatments recommended for PMS capable to partially bring relief, their side effects, however, have caused a great concern, shifting scientists’ attention towards the nutritional approach.^9,10^The role of calcium (Ca) and magnesium (Mg) in decreasing pain and alleviating the severity of symptoms is reported.^11,12^ Factors affecting PMS are both personal and environmental factors along with lifestyle all together can make considerable changes in health and quality of life of women suffering from PMS.^13^ PMS symptoms have also been linked to lower dietary intakes of some major food groups such as dairy products.^14^

 Lack of knowledge regarding the etiology of PMS is one of the most difficulties in some women resulting in the change of behavioral and emotional issues especially in the luteal phase which impress their healthy lifestyle.^15^ However, limited number of studies assessed the association of vitamin D with this condition.^15^ Emerging line of evidence have found decreased Ca levels during ovulation in relation to the luteal phase, while a number of reports revealed decreases in 25-hydroxyvitamin D levels during the span of the menstrual cycle itself.^16^ Consuming Ca supplement resulted in significant decreases in somatic symptoms such as headache, joint pain, and some emotional disorders like appetite changes, depression, and sleep disorders in women with PMS.^17^

 To our best knowledge, no study has assessed the relation of these related nutrients with PMS in young women before. The status of Ca, vitamin D and Mg has key role against PMS prevalence and due to health and medical consequences of the syndrome, this study was aimed at comparing these triad nutrients in young students suffering from PMS with apparently healthy young women.

## Materials and Methods

###  Participants and procedures

 This research was conducted as a case-control study on 62 female students (31 participants diagnosed with PMS and 31 matched participants as their controls) within the age range of 20-22 years residing in the dormitory at the Abadan University of Medical Sciences, Abadan, a city located by the Persian Gulf, South-West of Iran in 2015. To obtain the cases, we conducted a preliminary screening as described below.

 The sample size was calculated using a case-control equation for the comparison of two independent means in which initial number of the sample was obtained 23 participants in each group. To prevent possible dropouts, 62 participants were recruited in the two groups (each 31 participants).^18^

###  Ethical Considerations

 Research protocol was approved by the Medical Ethics Committee at the Jundishapur University of Medical Sciences, Ahvaz, Iran.

###  Inclusion Criteria

 First, a set of questions of Utah PMS Calendar II was used.^19^ Five common symptoms were selected and for each symptom 4 degrees (asymptomatic-mild-moderate and severe) were determined, the maximum interval for repeat, was 35 days. Then the questionnaire was distributed among 180 participants and they were asked to fill in the forms of two consecutive periodic cycles. Symptoms of those began from 10 days before menstruation and ending at the beginning of menstruation were recorded. The diagnosis was made by an attendant gynecologist.^20^ Participants were weighed at the first and second phase of periodic cycle. The participants did not receive any medicine in this study.

 The participants that had more than 5 symptoms (including depression, feeling disappointed, nervousness, insomnia, drowsiness, headache, breast tenderness, sweating) were regarded as PMS group. Participants in the same age range with less than four of the above mentioned criteria were considered as controls (n_1,2_=31).^21^

###  Exclusion criteria

 Exclusion criteria consisted of the past medical history of anxiety disorders, depression, untreated hypothyroidism, irregular menstruations or any changes in diagnosis of PMS within the last year. Those who were consuming Ca, Mg, vitamin D or herbal supplements were also excluded.

###  Measures

 To measure serum total Ca, Mg and Vitamin D, five mL fasting blood sample was taken. Blood samples were centrifuged and serums were collected. Serum concentrations of Ca and Mg were assessed using Pars Azmoon kit, Tehran, Iran according to instructions provided. Vitamin D serum levels were analyzed by ELISA method using IDS detection kit, Germany.

###  Statistics

 The nutritional status was analyzed using customized Nutritionist IV^®^ software. For comparing the results between the two group independent *t*-test, for nonparametric equivalent Mann-Whitney- U test and for qualitative variables chi-square test were applied (SPSS ver.18) (Chicago. IL, USA). For comparing the variables between the groups, the ANCOVA test was used.

## Results

 Totally, 62 students (31 PMS cases and the same number of healthy controls) were enrolled. There were no statistically significant differences in any of the basic characteristics between the two groups (Table 1).

 Serum concentrations of Ca and Mg were reduced significantly in PMS participants (*P*<0.05), but vitamin D_3_ levels did not show any significant differences between the groups.

**Table 1:** Basic Characteristics of the Participants

**Variables**	**Controls(n=32)**		**PMS(n=32)**		***P-*** **value** *
	**Mean**	**SD**	**Mean**	**SD**	
Age (yr)	20.6	1.3	21.1	1.2	0.412
Weight (kg)	55.3	7.8	52.8	7.2	0.232
Height (cm)	160.7	5.6	160.5	6.5	0.901
BMI (kg/m^2^)	21.4	2.4	20.5	3.1	0.233

* *t*-test was applied. Abbreviations: BMI, Body mass index; PMS, Premenstrual syndrome

 The risk of having PMS was increased by 19% and 14% for each unit decrease in serum Ca and Mg levels, respectively (*P*<0.05; Table 2).

**Table 2:** Serum levels of vitamin D, magnesium and calcium in all participants

**Variables**	**Controls**		**PMS**		**Odds Ratio** **(95% CI)**	***P-*** **value** *
	**Mean**	**SD**	**Mean**	**SD**		
Vitamin D (ng/mL)	6.66	3.75	6.84	4.07	0.96 (0.87 – 1.22)	0.817
Mg (mg/dL)	1.98	0.29	1.83	0.23	0.86 (0.72 – 0.93)	0.034
Ca (mg/dL)	9.52	0.38	9.46	0.43	0.81 (0.67 – 0.89)	0.017

Abbreviations: Ca, Calcium; Mg, Magnesium; PMS, premenstrual syndrome. *Mann-Whitney U-test was applied

 More than 85% of all participants showed degrees of vitamin D deficiencies and more than one-third of the PMS cases revealed Mg deficiencies (*P*<0.05; Table 3).

**Table 3:** Prevalence of Ca, vitamin D and Mg deficiency in participants

**Nutrients serum Levels**	**Control**		**PMS**		
	**Frequency**	**%**	**Frequency**	**%**	***P*** **-value**
Ca (mg/dL)					0.040
Deficiency<8.5	1	3.2	1	3.4	
Normal 8.5-10	30	96.8	27	93.2	
High>10	0	0.0	1	3.4	
Vitamin D (ng/mL)					0.823
Sever deficiency <10	10	32.3	9	32.1	
Mild-Mod deficiency 10-30	18	58.1	15	53.6	
Normal 30-70	3	9.7	4	14.3	
Mg (mg/dL)					0.031
Deficiency<1.8	7	22.6	10	34.5	
normal1.8-3	24	77.4	19	65.5	

* Chi square test was applied

 Comparison of nutrients intake revealed significant differences for Ca, Mg and potassium intakes between the groups (Table 4). The intake of vitamin D was not reliable due to insufficient dietary resources.

**Table 4:** Selected daily nutrients Intake of all participants

**Nutrients**	**Controls**		**PMS**		***P-value***
	**Mean**	**SD**	**Mean**	**SD**	
Energy (kcal)	1838.5	218.6	1918.06	196.7	0.113
Protein (g)	52.9	8.6	56.71	9.7	0.627
Fat (g)	82.3	11.74	88.62	15.4	0.637
Saturated Fat (g)	16.6	3.82	18.27	5.4	0.164
PUFA (g)	25.4	4.67	26.38	4.9	0.137
MUFA (gr)	34.8	5.27	37.5	6.96	0.702
Carbohydrate (g)	228.25	40.38	228.67	34.1	0.084
Iron (mg)	9.6	1.67	10.51	1.5	0.164
Zinc (mg)	6.9	1.12	7.62	1.2	0.469
Copper (mg)	1.1	0.25	1.07	0.2	0.143
Magnesium (mg)	210.5	43.36	218.7	44.5	0.036
Potassium (mg)	1811.2	354.34	1697.6	247.19	0.045
Calcium (mg)	438.6	118.42	421.9	116.71	0.047
Vitamin D (mcg)	2.9	14.64	4.68	16.57	0.808
Vitamin A (IU)	551.6	419.46	531.5	446.55	0.857
Vitamin E (mg)	26.8	5.90	27.75	6.21	0.675
Thiamin (mg)	1.3	0.28	1.33	0.26	0.685
Riboflavin (mg)	.9	0.22	.87	0.20	0.877
Niacin (mg)	16.5	3.40	16.8	3.28	0.028
Pyridoxine (mg)	1.4	0.42	1.38	0.34	0.894
Cobalamine (mcg)	1.82	0.66	2.07	0.77	0.864
Vitamin C (mg)	63.9	23.36	52.41	26.62	0.5
Cholesterol (mg)	148.9	63.85	184.1	86.99	0.08
Dietary Fiber (g)	13.87	3.23	13.7	2.96	0.3
Sugar (g)	47.77	17.98	36.4	15.35	0.8

## Discussion

 PMS is one of the health problems in females' reproductive age and many of them are resulted in physical and psychological symptoms and also this condition may have adverse effects on lifestyle.^1,2^

 Our study evaluated the food intake and serum levels of Ca, Mg and vitamin Din University students with PMS compared to those of the healthy matched controls. We found no statistically significant difference between the PMS cases and their healthy counterparts regarding the vitamin D serum levels. However, Ca and Mg serum concentrations were lower in the cases although all were within the normal range.

 The study of Bendlich et al. showed lower levels of Ca in those without PMS and that an increase in dietary Ca intake could help maintain the normal levels and prevent the manifestations of the condition.^8^ The possibility of the role of low serum Ca in PMS is reported.^11^ Consumption of milk and dairy products was associated with decrease in other complications such as acne and increased weight and the authors concluded that appropriate food pattern is recommended for PMS participants.^17^

 In a study, 1000 mg of Ca prescribed daily to those with PMS has led to a 61% reduction of physical and 62% reduction of psychological symptoms,^17^ and confirmed the fact that hypocalcaemia could be a contributing factor in PMS.^17^ Previous studies have also reported similar results.^22,23^ Such results could be biased by intake of supplements containing Ca, vitamin D or other multivitamins and mineral compounds.^15^ However, this is not the case in our participants.^15^ In our study, the amount of Ca intake in both PMS and the controls were less than half daily recommended intake (DRI). The serum vitamin D_3_ levels of PMS cases were not different from those of their controls, which were in agreement with Berton-Johnson et al.^15^ Other than Ca, minerals such as K and Mg also vary during the menstruation cycle.^24^ Although Posaciet al., have reported low Mg levels in those with PMS.^25^ Khine et al. have found no differences in Mg serum levels when slow releasing Mg supplements or placebo were used in PMS participants.^26^ In our PMS participants both serum levels and dietary intakes of Mg were lower than their healthy counterparts. Furthermore, about 34% of cases with MS vs. 23% of the controls showed low Mg serum levels.

 Although our results are not in agreement with those of Khine et al.^26^ however, they are in the line with Posaci et al., findings.^25^A more recent study, did not report any effects of Mg in reducing the symptoms of PMS.^27^

 Unlike Bakhshani et al.,^28^ we did not find any differences regarding the intake of other nutrients between the study groups except potassium and niacin, which were not of clinical significance.

 For obtaining more precise results, we excluded several intervening variables such as history of anxiety disorder, depression and irregular menstruations during the last year and untreated hypothyroidism that could interfere with the results.

 As a strength point of our study, it merits mentioning that there has been no case-control study designed to assess the triad synergistic nutrients of vitamin D, Ca and Mg so far and these nutrients have been an area of interest in the recent years due to their worldwide deficiencies considered as major health problems and also their newly defined functions.

 As a limitation of our study, we used a 24-hour recall questionnaire, which may result in incomplete dietary intake. Moreover, our participants were living in the university dormitories in which self-service meals were served, and this could have affected their routine dietary habits.

## Conclusion

 The participants had no differences in demographic data. In addition, they had no differences in disease severity and the duration of periodic cycle. Our study indicates that there are lower serum levels of Ca and Mg in PMS participants than their healthy controls while no difference is evident in terms of vitamin D serum status. Moreover, participants with PMS had lower dietary intakes of Ca and potassium. Based on our findings, nutritional recommendations should be made to improve the quality of diet encouraging intake of good sources of Ca, Mg and K in subjects with PMS. Regarding the health consequences of vitamin D, calcium and magnesium deficiencies and wide prevalence of PMS, population studies are warranted to explore the impact of improving nutritional status on symptoms of PMS.

## Acknowledgement

 The costs of laboratory tests were provided by Vice-Chancellor for Research at the Jundishapur University of Medical Sciences.

 Authors would like to thank Dr Khazenifar and Dr Rezaei who helped with diagnosis the PMS cases.

## Competing interests

 The authors claim no competing interests.
