# The association between the risk of premenstrual syndrome and vitamin D, calcium, and magnesium status among university students: a case control study [Health Promotion Perspectives, 2015, 5(3), 225-230]

**DOI:** 10.15171/hpp.2016.09

**Published:** 2016-03-31

**Authors:** Afsaneh Saeedian Kia, Reza Amani, Bahman Cheraghian

**Affiliations:** ^1^International Arvand Medical Sciences University, Abadan, Iran; ^2^Department of Nutrition, Diabetes Research Center, Jundishapur University of Medical Sciences, Ahvaz, Iran; ^3^Department of Epidemiology, Jundishapur University of Medical Sciences, Ahvaz, Iran

**Keywords:** Premenstrual Syndrome, Calcium, Magnesium, Vitamin D

## Abstract

**Background:** Premenstrual syndrome (PMS) is one of major health problems in childbearing age women. Herein, we compared the nutritional status of vitamin D, calcium (Ca) and magnesium (Mg) in young students affected by PMS with those of normal participants.

**Methods:** This study was conducted on 62 students aged 20‒25 yr in the city of Abadan (31 PMS cases and 31 controls). All participants completed four or more criteria according to the Utah PMS Calendar 3. Age, height, body mass index (BMI), serum Ca, Mg and vitamin D levels and a 24-hour food recall questionnaire were recorded.

**Results:** Vitamin D serum levels were lower than the normal range in the two groups. The odds ratios (CI 95%) of having PMS based on serum Ca and Mg concentrations were 0.81(0.67 – 0.89) and 0.86 (0.72 – 0.93), respectively. Based on serum levels, 85% of all participants showed vitamin D deficiencyand more than one-third of the PMS cases were Mg deficient (P<0.05). In addition, there were significant differences in dietary intake of Ca and Mg, and potassium but not vitamin D in the two groups. Dietary intakes of Ca and Mg were quite below the recommendation in all participants.

**Conclusion:** Vitamin D, Ca and Mg nutritional status are compromised in PMS subjects. Because PMS is a prevalent health problem among young women, it merits more attention regarding improvement of their health and nutritional status.

In this original article, a typo error was detected in the abstract: Results section, third line, “85% of all participants showed vitamin D deficiency” is correct, which was published as: “855 of all participants showed vitamin D deficiency”.

The online version of this review paper is accessible in doi:10.15171/hpp.2015.027

**ePublished:** 31 Mar. 2016

